# *Aeonium decorum* as a microbial recruitment platform for atmospheric polycyclic aromatic hydrocarbons mitigation in urban gardens

**DOI:** 10.1186/s40793-026-00914-7

**Published:** 2026-05-31

**Authors:** Felix Velando, Lázaro Molina, Irene Hurtado, Pieter van Dillewijn, Ana Segura

**Affiliations:** Environmental Protection Department, Estación Experimental del Zaidín, Consejo Superior de Investigaciones Científicas, C/ Profesor Albareda 1, 18008 Granada, Spain

**Keywords:** Atmospheric contaminants; naphthalene; bioremediation, Biodiversity, Plant-bacteria associations

## Abstract

**Background:**

In the context of the Sustainable Architecture, green roofs, green walls, green belts or urban farms are becoming popular infrastructures in cities and have been proposed as promising elements to ameliorate air pollution. Atmospheric contaminants are deposited not only on the foliar surface of plants, but also in soils. Plants may interact with pollutants, but their associated microbiomes (epiphytic, endophytic and rhizospheric) may harbor contaminant-degrading bacteria which could play an important role in pollutant mitigation. Therefore, we explored the effects of atmospheric contaminants, using naphthalene as a model compound, on some of the living elements of urban gardens (plants and microbiomes).

**Results:**

Exposure to gaseous naphthalene had weak effects on *Aeonium decorum* and *Trifolium repens* plants (measured as efficiency of photosystem II), and on soil bacterial diversity. Although the presence of naphthalene is not the major driver of soil bacterial community structure, metagenomic and qPCR analysis revealed an increase in polycyclic aromatic hydrocarbon (PAH)-ring hydroxylating dioxygenases in *Aeonium* planted soils, suggesting a positive effect of this plant species for the selection of potential contaminant-degrading microbes. We have also observed an increment in *Pseudomonas* (known for their capacity to degrade contaminants) and *Solimonas* in response to naphthalene. Validation of tools designed to evaluate the exposure of plants to atmospheric contaminants was performed creating urban gardens planted with *A. decorum* plants and exposed to environmental conditions.

**Conclusions:**

Our results suggest that *Pseudomonas* and *Solimonas* could be used as markers for biodegradation. *A. decorum* is proposed as a good candidate for amelioration of atmospheric contaminants and gardens constructed with these plants carried PAH degrading bacteria on leaf surfaces indicating that they have the capacity to respond to the presence of contaminants.

**Supplementary Information:**

The online version contains supplementary material available at 10.1186/s40793-026-00914-7.

## Background

Increasing human population and its concentration in urban areas have created a great pressure for for land urbanization, with the construction of taller and larger buildings leading to the emergence of new megacities and the decreased of green areas. Currently, 55% of the world population lives in urban areas and, by 2050, it is expected to increase to 68% [1]. The expansion of urban areas, combined with the current consumption model, which is mainly based on the utilization of fossil fuels, has significantly increased air pollution in cities, posing health risks to humans animals and plants [2, 3]. Air pollution causes approximately seven million deaths annually (World Health Organization [WHO], 2021). In response to growing concerns about indoor and outdoor air pollution, national and international environmental Agencies are enforcing policies to reduce emissions, and promote active strategies to mitigate the impact of atmospheric contaminants. Within this framework, polycylic aromatic hydrocarbons (PAHs) and volatile organic compounds (VOCs) are now considered as re-emerging contaminants [4]. Fine particulate matter (PM_2.5_) represents almost 50% of atmospheric PM iemissions, and its toxicity has been partially attributed to its high content of PAHs, BTEX, PCBs (polychlorinated biphenyls) and PCDD/Fs (polychlorinated dibenzo-*p*-dioxins and dibenzofurans) [5]. It is estimated that almost one-third of atmospheric PAHs emissions are produced during the combustion of fossil fuels, with naphthalene and its derivatives being the most abundant [6, 7].

Sustainable Architecture, based on the use of renewable and recycled resources and aiming to reduce the construction carbon footprint, was originally conceived to harmonize urban life with nature, promoting energy conservation and minimizing long-term environmental damage [8–10]. Integrating plants into urban infrastructure offers additional benefits for mitigating the damaging effects of pollution [11–14]. Green roofs, green walls, green belts and urban farms are becoming popular infrastructures in urban landscape.

Particulate matter is deposited not only on the foliar surface, but also in soils. Plants can adsorb, absorb and metabolize various contaminants and their associated microbiomes (epiphytic, endophytic and rhizospheric) can contain contaminant-degrading bacteria [15–17]. Thus, plants and their associated microbiomes are expected to play a central role in pollutant mitigation. However, high concentrations of contaminants can hinder plant growth decreasing the rate of contaminant elimination. Many of the green infrastructures have a high degree of isolation, and have been considered as new ecosystems that differ from natural environments, Therefore, knowledge about how microbial communities and plants within the green infraestructures respond to atmospheric contaminants will help optimize the design of efficient urban infrastructures to mitigate pollutants that severely impact human health. The ecological roles of plant–microbe consortia in mitigating airborne contaminants remain insufficiently explored in these specific environments.

This study addresses this knowledge gap by investigating how plant and microbial communities within green infrastructures respond to atmospheric pollutants, using naphthalene as a model contaminant. Naphthalene is a semi-volatile PAH that sublimes at room temperature. It is produced mainly during the combustion of fossil fuels, but it is also used as intermediate in the production of phthalic anhydride, surfactants and pesticides. Among the different sources of airborne naphthalene are industries, traffic, some heating systems, and forest fires. Indoor naphthalene sources include deodorizers, repellents and fumigants and cigarrettes. Short-term symptoms of exposure to naphthalene fumes include headache, eye irritation, nausea, and anemia, while chronic exposure may cause damage to the respiratory system, eyes, skin, kidney, liver, and nervous system [18, 19]. It has also been classified as possibly carcinogenic to humans (Group 2B) by the International Agency for Research on Cancer [20]. Average atmospheric naphthalene concentrations may range from 0.18 to 1.7 µg m^−3^ in non-smokers homes and from 0.02 to 0.94 µg m^−3^ outdoors in urban areas [6], and are generally below the potential hazardous limits [i.e. RfC (Reference concentration for inhalation exposure; US-EPA): 3 µg m^−3^; or LCI (lowest concentration of interest for VOC emissions from building products; AgBB) = 10 µg m^−3^] [21]. However, there are wide differences among different locations [22, 23] and in many assessments naphthalene ranks at or near the top of those substances posing inhalation cancer risks [22]. Naphthalene is also phytotoxic, causing oxidative stress in plants and decreasing biosmass [24]. The aim of this study is to facilitate protocols for the straightforward design of urban infrastructures elements aimed to advance optimized phytoremediation strategies. To this end, we set-up a semi-isolated system in which naphthalene is the sole atmospheric contaminant and we have characterized the responses of plants and microbes, as well as the dissipation of naphthalene under these conditions. Subsequently, we evaluated the responses of simulated roof-gardens with the best-performing plants identified in the previous experiments under atmospheric conditions. By characterizing the biological responses of plants and microbes to naphthalene exposure and under real conditions, we expected to gain insights into plant-microbes interactions during VOC remediation to help improve air quality in cities.

## Methods

### Naphthalene elimination

*Aeonium decorum* and *Sedum album* were selected as succulent plants while *Lavandula angustifolia* and *Euonymus japanicus* were chosen due to their frequent use in gardens in Granada because their low water requirements. These plants grown in universal substrate were obtained from a nursery in Gójar (Granada, Spain). Grass and clover were selected as herbaceous plants; in either case seeds were sown 15 days prior to the assay.

Pots were kept overnight in a tray with water for soil to reach 100% water holding capacity. Subsequently, pots with or without plants were placed inside a 5 L glass jars, each containing an open vial with 50 mg of solid naphthalene (Merck, S.L.U) or an empty vial (control). The jars were closed with lids and kept outdoors in an umbracle under environmental conditions for 15 days. Experiments were conducted in November—December 2023 except for clover which was tested in December 2024. According to the meteorological station at Granada Airport, the mean temperature was 11 °C in November 2023 (min:—2.5 °C, max: 27.7 °C), 7 °C in December 2023 (min: 0.1 °C, max: 15.6 °C) and 7 °C. In December 2024 (min:—4.5 °C; max: 21.2 °C). The autumn season was selected because diurnal temperatures were high enough to allow the active growth of the plants. Under these conditions, it was estimated that in an airtight 5 L container, the quantity of sublimated napthalene at equilibrium would range between 1.17 and 0.75 µg; therefore the gaseous naphthalene in our systems should be lower than these values. For systems without plants, pots in which the aerial part of the plant had been removed were used to ensure that the type of soil did not differ from that of planted systems. Because *Trifolium repens* (clover) is a model system in our laboratory and because herbaceous plants have been reported to be effective for rhizoremediation due to their extensive root system, we tested the performance of clover in naphthalene dissipation. Clover seeds were sown in Universal Substrate Premium, Substralomb, (Serrano Elvira CB, Alhendín, Granada) 2 weeks before the start of the experiment and 25 mg of naphthalene were used in the treatments. Clover control systems consisted in pots with unplanted bulk soil from the same source.

Fifteen days after naphthalene addition, the systems were opened, and the remaining naphthalene was dissolved in 5 mL methanol and quantified by HPLC. HPLC was performed using an Agilent 1260 Infinity II system (Agilent Technologies, Santa Clara, CA, USA) equipped with a diode array detector (DAD) and a Waters (Milford, MA, USA) Nova-Pak C18 column (4 µm, 3.9 mm × 150 mm). The mobile phase consisted of acetonitrile (ACN) and MilliQ water both acidified with 0.1% orthophosphoric acid. Samples were run isocratically for 9 min at a flow rate of 0.85 mL/min with a 65% (v/v) ACN:water ratio. Detection was set at 275 nm.

### Imaging-PAM experiments

To measure the maximal quantum yield of photosystem II (PSII), imaging PAM (Pulse Amplitude Modulation) fluorometry was used. *A. decorum* plants were removed from the closed systems and dark-adapted for 20 min. A high-intensity saturation pulse (measuring light intensity: 2, frequency: 4, saturation pulse intensity: 10) was applied to determine maximum fluorescence yield (Fm). The maximum PSII quantum yield (Fv/Fm) was calculated using the formula:

$$ Fv/Fm \, = \, \left( {Fm \, - \, F0} \right) \, / \, Fm, $$

where F0 is the minimum fluorescence of the dark-adapted sample.

Statistical analyses were performed using Sigma Plot 11.0 software. Differences were tested using a *t*-test with a significance threshold of *p* < 0.05.

### Soil DNA extraction and qPCR analyses

Soil samples were collected and frozen at −20 °C before DNA extraction. which was performed using the Fast DNA Spin Kit for Soil (MP Biomedicals LLC, Solon, Ohio, USA) following manufacturer´s instructions. DNA purity and concentration were determined using Nanodrop ND-1000 (Thermo Fisher Scientific) and Qubit dsDNA BR Assay kit (Live Technologies, Invitrogen, USA).

To estimate total bacteria abundance, primers 341F and 534R targeting the 16S rRNA gene were used [25]. For estimation of the number of PAH-ring hydroxylase dioxygenase genes, oligonucleotides PAH-RHDα-GN-F and PAH-RHDα-GN-R were used [26]. Quantitative qPCR assays were performed using 1 ng of soil DNA mixed with SYBR™ Green PowerUp™ master mix (Fisher Scientific) and the appropriate oligonucleotides. Reactions were run on a LightCycler 480II Real-Time PCR instrument (Roche Diagnostics Ltd, Rotkreuz, Switzerland) with 35 cycles (95° for 30 s, 65 °C for 30 s and 72 °C for 30 s).

To quantify 16S rRNA molecules, qPCR was performed using serial dilutions of genomic DNA from *Novosphingobium resinovorum* HR1a, quantified with a Nanodrop ND-1000. For quantification of PAH-RHDα of gram-negative bacteria, a fragment of the PAH-RHDα gene of *Burkholderia* sp. MS3 was amplified using the PAH-RHDα-F and PAH-RHDα-R oligonucleotides and genomic DNA as template. The band was extracted from an agarose gel and, quantified, and serially diluted for qPCR analysis. Statistical analysis were performed using the SigmaPlot 11.0.

### Bacterial diversity analysis

DNA from soil was obtained with the Fast DNA Spin Kit for Soil (MP Biomedicals LLC, Solon, Ohio, USA) as described above. Paired-end amplicon sequencing of the V3-V4 region of the 16S ribosomal RNA gene was performed using primers 5’ CCTAYGGGRBGCASCAG 3’ and, 5’ GGACTACNNGGGTATCTAAT 3’ by Novogene Co, Ltd (Cambridge, United Kingdom) on and Illumina platform, generating 250 bp paired-end reads. Data was provided as forward and reverse reads with barcodes and primer sequences removed. Four independent biological replicates were sequenced and analyzed.

Raw 16S rRNA gene amplicon sequences were processed using the DADA2 pipeline [27] in R (v 5.4.0). Primer sequences were removed using cutadapt, and reads were quality-filtered, dereplicated, and denoised using the filterAndTrim, learnErrors, derepFastq, and dada functions. Paired-end reads were merged, and chimeric sequences were removed using removeBimeraDenovo. Amplicon sequence variants (ASVs) were inferred with single-nucleotide resolution. Taxonomic assignment was performed using the assignTaxonomy function with the SILVA reference database (v138).

The resulting ASV table, taxonomy table, and sample metadata were integrated into a phyloseq object using the phyloseq R package [28]. Samples with low sequencing depth were excluded from downstream analyses. To account for differences in sequencing depth across samples, ASV counts were normalized to relative abundances using the transform_sample_counts function in phyloseq prior to diversity estimation. Alpha diversity metrics, including Observed Richness, Shannon Index, and Simpson Index, were calculated using the estimate_richness function in phyloseq. Diversity comparisons between experimental groups were visualized using ggplot2, and statistical significance was assessed using non-parametric Kruskal–Wallis tests.

For beta diversity analysis, ASV counts were normalized to relative abundances using transform_sample_counts. Bray–Curtis dissimilarity matrices were computed using the distance function. Bray–Curtis was selected because it is robust for ecological count data and is one of the most widely used dissimilarity metrics in microbiome studies, providing a reliable measure of compositional differences among samples. Ordination was performed via Principal Coordinates Analysis (PCoA) using the ordinate function. Group clustering and separation were visualized with plot_ordination. Permutational multivariate analysis of variance (PERMANOVA) was conducted using the adonis2 function from the vegan package to test for significant differences in community composition across treatments [29].

### Metagenome analysis

For metagenome analysis, three independent biological replicates were analyzed. DNA from soil was extracted as described above and submitted for shotgun metagenomic analysis to Novogene Co, Ltd (Cambridge, United Kingdom). There, DNA was randomly sheared, end-repaired, A-tailed, ligated with sequencing adapters, size selected, and purified prior paired-end 150 bp sequencing on an Illumina NovaSeq X Plus Series.

After quality filtering, reads were assembled using MEGAHIT [30] and Open Reading Frame (ORF) prediction on the resulting scatigs was performed using MetaGeneMark [31]. ORF dereplication was performed with CD-HIT [32] and gene abundance was quantified using Bowtie2 [33]. Comparison of Unigenes to the KEGG functional database [34] was performed using DIAMOND [35].

### Assesing bacterial diversity and PAH-associated microbes in *Aeonium* artificial gardens systems

For the preparation of artificial gardens, we planted *Aeonium* plants in three 25 L rubber buckets (ten plants per bucket) filled with Universal Substrate Premium Substralomb (Serrano Elvira CB, Alhendín, Granada) and we left them outdoors for 6 months (October-24–April-25) under environmental conditions at different locations within the Estación Experimental del Zaidín (Granada, Spain).

To determine the presence of PAHs, 18 leaves collected from each garden after 6 months outdoors were swabbed with the same cotton swab previously moistened with methanol to collect deposited dust. The hydrophobic compounds (including PAHs) were extracted from each swab by adding 2 mL methanol, vortexing for 1 min, and centrifuging for 2 min. The resulting extracts were analysed by HPLC as described above, except 53% (v/v) ACN:water mobile phase was used for isocratic runs of 24 min, with detection at 230 nm. This allowed the detectiion of PAHs with up to four aromatic rings, including naphthalene, phenanthrene and pyrene.

One leaf of four different plants from each of the three buckets were collected at the start of the experiment and stored at −20 °C. After 6 months outdoors, six leaves from three different plants from each bucket were collected. The twelve leaves collected at the start of the experiment or the 18 leaves collected in each garden after 6 months oudoors, were introduced into 100 mL flasks with 10 mL of M9 minimal media and flasks were shaked for 1 h to resuspend leaf-associated bacteria. After agitation, the leaves were removed from each suspension and then centrifuged. DNA was extracted from the resulting pellets using the Wizard® Genomic DNA Purification Kit (Promega). Three replicate soil samples were also collected at the start of the experiment and three from each garden after 6 months, and DNA was extracted as described above. DNA from leaf surfaces and soil was sequenced and analyzed as decribed above for Bacterial Diversity Analysis.

To evaluate the capacity of the *Aeonium* leaves to support bacteria capable of degrading contaminants, we carried out an enrichment of epiphytic bacteria in minimal media with phenanthrene as the sole carbon source as described by Segura et al. (submitted for publication in the *International Journal of Systematic and Evolutionary Microbiology*). Briefly, *Aeonium* leaves from nursery grown plants not used for gardens or other experiments were cut and introduced into a 100 mL flask with 10 mL of M9 minimal media. After 16 h of agitation, leaves were removed, 1 mg of phenanthrene was added, and the culture incubated at 30 °C with shaking for 2 days. One mL of this enrichment was used to inoculate a flasks with fresh media and phenantrene. The enrichment procedure was repeated twice before serial dilutions were streaked one separate plates for purification if single colonies.

## Results

### Closed systems with *Aeonium decorum* plants eliminated more naphthalene than systems with other plants.

**Fig. 1:**
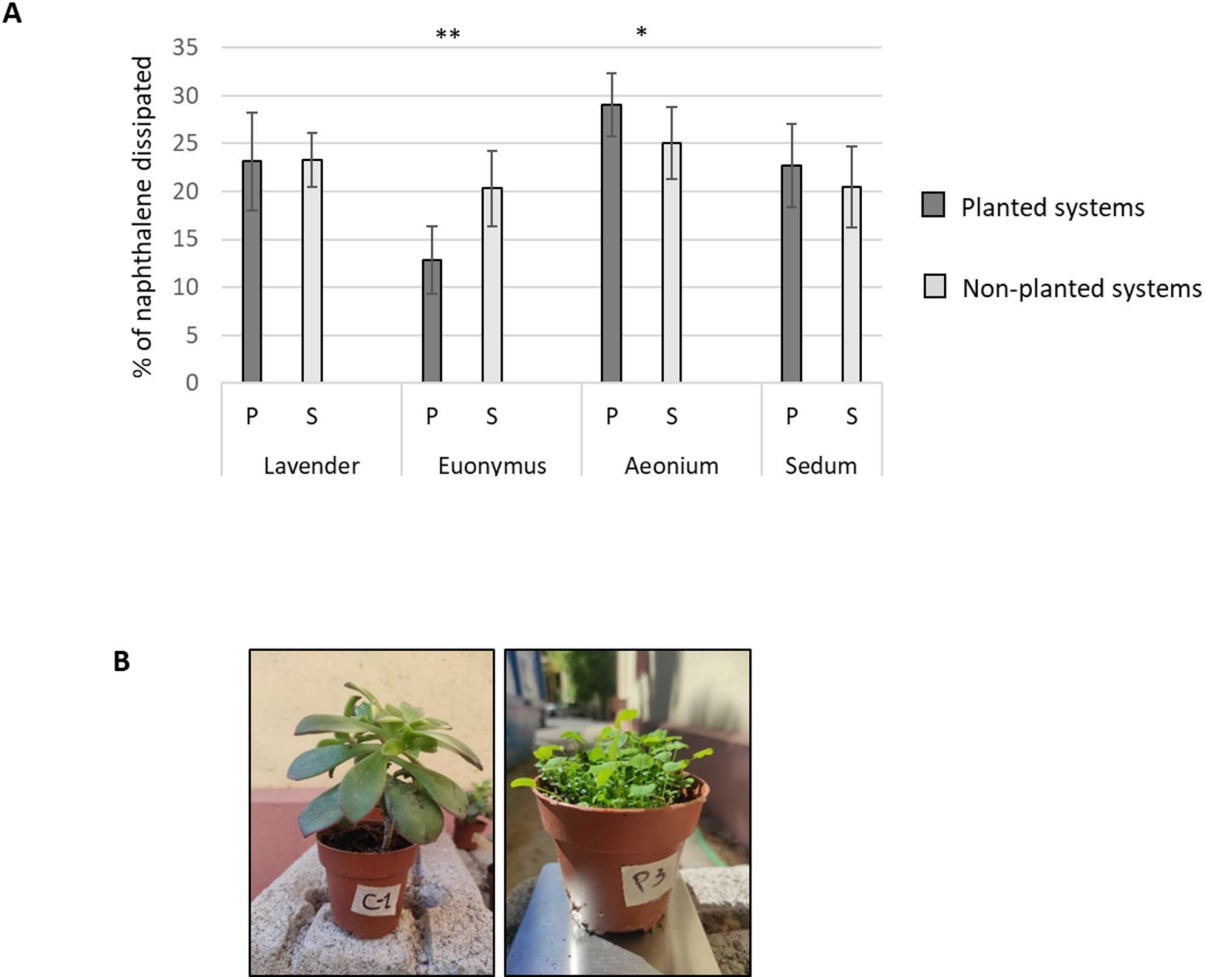
(**A**) Percentage of naphthalene removed by plants (lavender: *Lavandula angustifolia*; Sedum: *Sedum morganianum*; Euonymus: *Euonymus japonicus*; Aeonium: *Aeonium decorum*. Dark grey (P), indicates naphthalene removal in pots containing the whole plant; light grey (S) indicates naphthalene removal in experiments in which the aerial part of the plant had been eliminated. * *p* < 0.05; **p < 0.005. n = 8. (**B**) *Aeonium* (left) and clover (right) pots used in the experiments; plastic pots were of similar size

We explored different ornamental plants, typical of the Mediterranean climate, to assess the elimination of the gaseous polycyclic aromatic hydrocarbon, naphthalene. When we compared the systems with or without plants, only when using *Aeonium decorum* we detected a significant increase in the percentage of naphthalene dissipated (Fig. 1A). While in plant systems 14.5 ± 1.65 mg of naphthalene was dissipated (approx. 29% of the naphthalene added in the system), in systems in which the aerial part of *Aeonium* had been removed only 12.54 ± 1.86 mg (approx. 25%) of the volatile compound was dissipated. Therefore, we used this plant species for further experiments.

We observed around 13% naphthalene removal in clover systems and there were no statistically significant differences between planted and unplanted systems (clover systems removed 3.49 ± 0.63 mg and control systems 3.30 ± 0.34 mg). In conclusion, naphthalene dissipation was much lower using clover plants than *A. decorum* plants; as adsorption to the materials in the clover systems (plastic, soil), and gas interchange (our system is not hermetically closed) were similar for both plant experiments, the low amount of naphthalene dissipated with clover may be caused by the different biomass of the plants within the systems (Fig. 1B) and/or differences in biodegradation processes.

### Plants were not severely injured by the presence of gaseous naphthalene

**Fig. 2:**
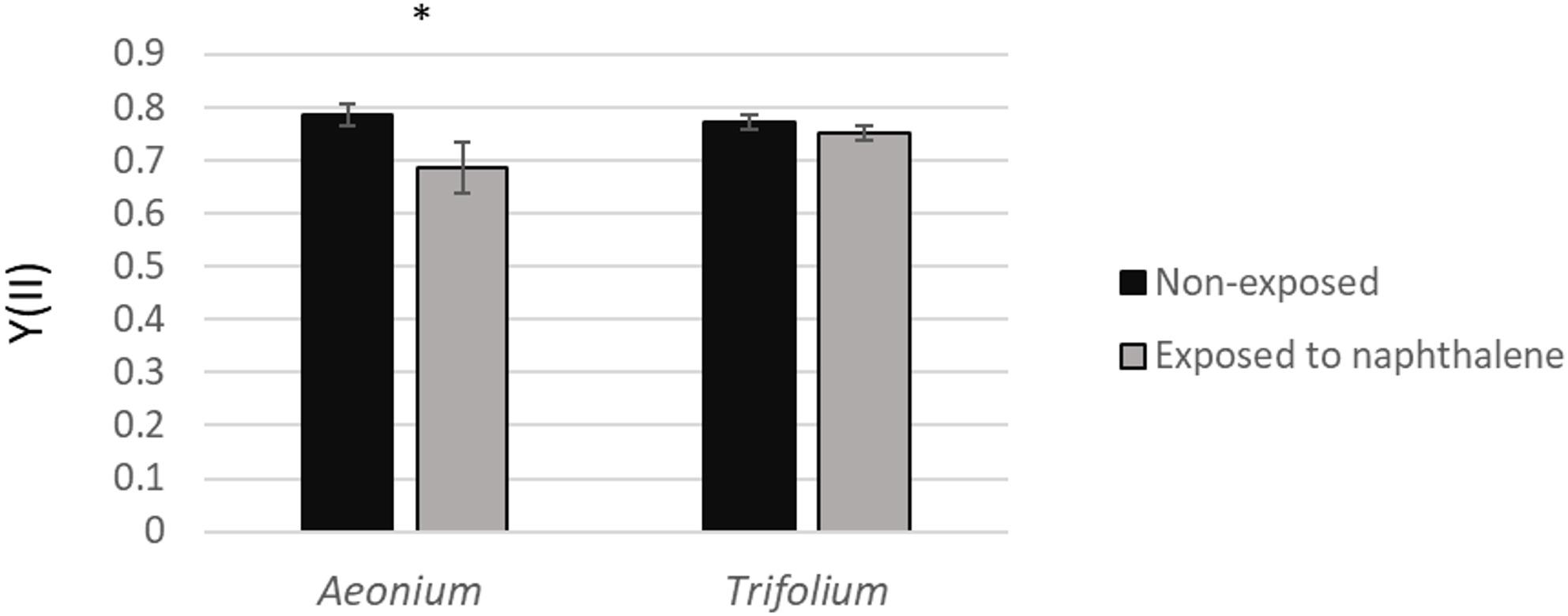
Effective quantum yield [Y(II)] in dark-adapted *Aeonium* and clover plants; Asterisk indicated statistically significant differences (*p* < 0.05)

Naphthalene is a toxic compound and, at certain concentrations, provokes severe effects in plants [36, 37]. However, at the concentrations used, we did not observe any of the visible symptoms reported in the literature (inhibition of growth, wilting, chlorosis or plant death) between non-exposed and naphthalene-exposed plants. To further analyze if plant physiology was altered by the presence of gaseous naphthalene, we used pulse-amplitude-modulated (PAM) fluorometry to study the state of the photosystem II under the two conditions (with and without naphthalene). We observed that the maximal PSII quantum yield of the dark-adapted *Aeonium* plants in control conditions (non-exposed to naphthalene) was 0.79 ± 0.02, while in dark-adapted plants treated with naphthalene this parameter was statistically significantly lower, 0.69 ± 0.05 (*p* < 0.05), suggesting that non-exposed plants were more efficient in converting absorbed quanta into chemically fixed energy (Fig. 2). Clover plants were minimally affected by the presence of naphthalene; the maximal PSII quantum yield of the dark-adapted non-exposed clover plants was 0.77 ± 0.01 while in dark-adapted plants exposed to naphthalene was 0.75 ± 0.01 (Fig. 2).

These results suggest that, under the conditions of the study, gaseous naphthalene was sensed by *A. decorum* plants but that the concentrations used in our assays were not high enough as to cause severe damage that could negatively influence their bioremediation effectiveness.

### Naphthalene has an impact on overall bacterial diversity in the *Aeonium* systems

**Fig. 3:**
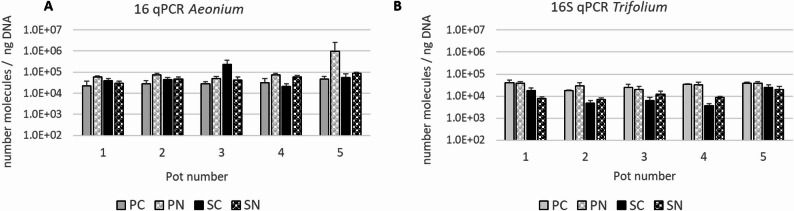
16S rRNA gene detection in (**A**) *Aeonium* experiments and (**B**) Clover experiments. PC: planted soil; PN: planted soil exposed to naphthalene; SC: control soil; SN: soil exposed to naphthalene. Bars represent the mean value of three qPCR replicates with error bars indicating the standard deviation. Results from 5 independent experiments are shown

We carried out an estimation of the number of bacteria in the different set-ups by using qPCR amplifying the 16S ribosomal RNA gene [25]. The number of bacteria were slightly higher in pots of *Aeonium* than in pots with clover. The estimated number of bacteria in *Aeonium* experiments were similar regardless of the treatment except for soils with plants treated with naphthalene in which the number of 16S rRNA gene molecules was statistically higher (*p* = 0.008) (Fig. 3A). In experiments with clover (Fig. 3B), pots with plants had higher numbers of bacteria than experiments without plants (PC vs. SC *p* = 0.012; PN vs. SN *p* = 0.001). As mentioned above, the experimental set-ups were different; while in *Aeonium* experiments, samples marked as soil were obtained from pots in which the aerial part of the plant had been removed, in clover experiments, soil samples came from unplanted soil.

**Fig. 4:**
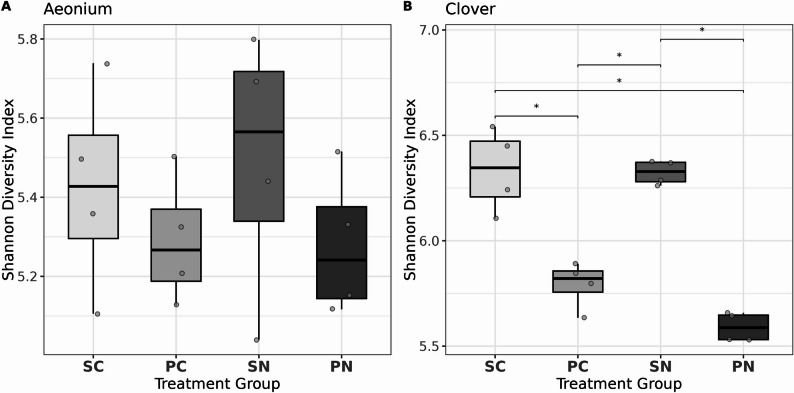
Changes in α-diversity (Shannon index) in closed systems using (**A**) *Aeonium decorum* and (**B**) clover*.* SC: control soil; PC: planted soil; SN: soil exposed to naphthalene; PN: planted soil exposed to naphthalene. The time of exposure to naphthalene was 15 days. The figure summarizes the results from four independent experiments. * *p* < 0.05

Bacterial diversity, studied by 16S rRNA amplicon sequencing analyses, showed no significant differences in α-diversity between samples with or without naphthalene in either *Aeonium* with and without the aerial part of the plant (Fig. 4 and Suppl. Table 1). However, in *Trifolium* experiments plants had a significant influence on α- diversity indexes and, in the case of bacterial richness (Observed) the presence of naphthalene had a significant effect (Fig. 4 and Suppl. Table 1).

The β-diversity PCoA analysis showed a clear clustering of samples from non-planted soil and clover-planted samples, while in *Aeonium* samples no significant differences were observed (Suppl. Fig. 1; Suppl. Table 2).

From these results, we could infer that the presence of clover decreased α-diversity and that the “plant effect” over β-diversity was more significant in clover than in *Aeonium* set-ups, probably because of the above mentioned differences between non-planted control experiments. However, exposure to gaseous naphthalene only had a significant effect on bacterial richness with clover but not with other α-diversity indices.

### Bacterial genera and genes related with naphthalene degradation were significantly more abundant in samples with naphthalene than samples without

**Fig. 5:**
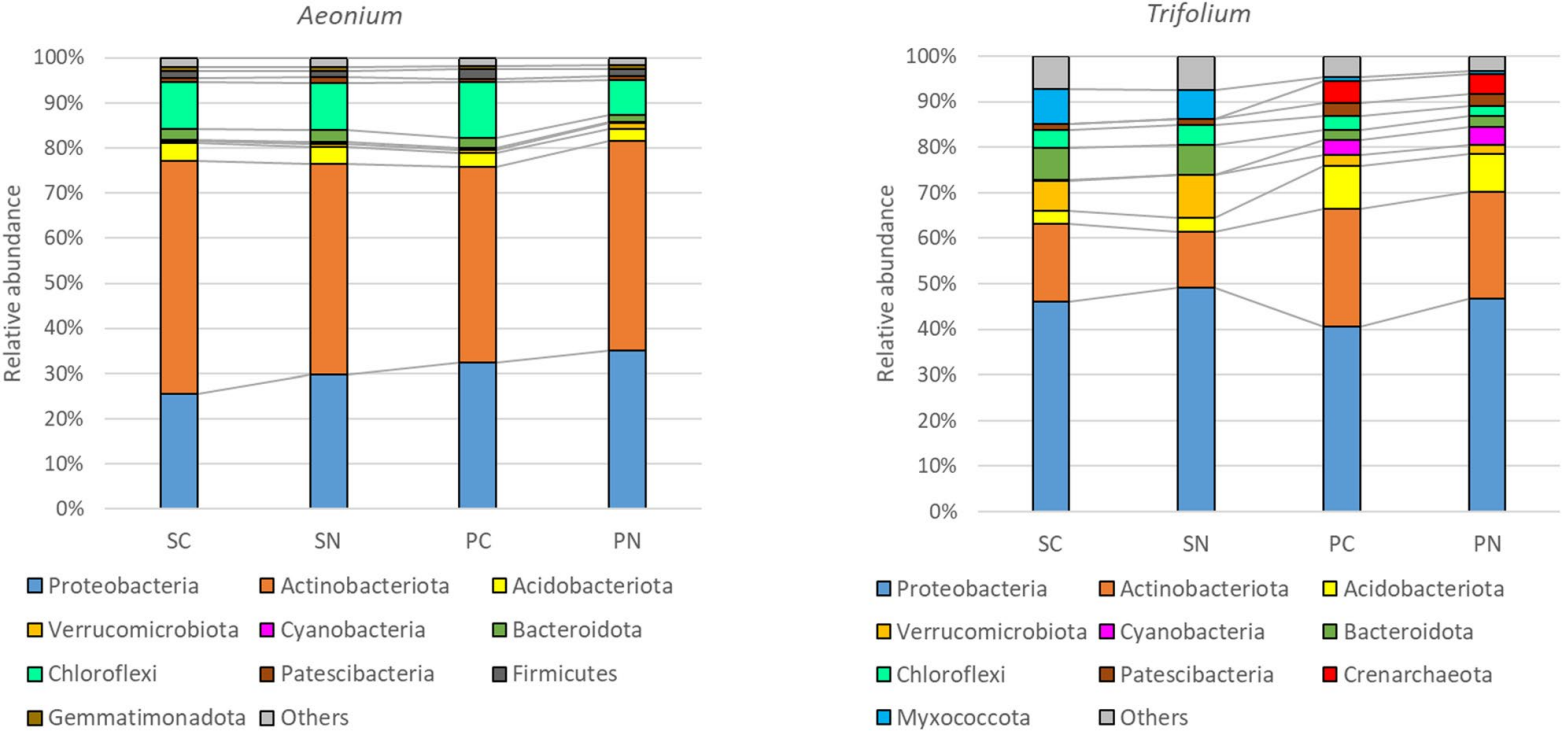
Relative abundance at Phylum level in soil samples taken from *Aeonium* experiments (left) and *Trifolium* experiments (right). SC: soil from non-planted experiments or without the aerial part of the plant; PC: soil from experiments with plants; NS: soil from non-planted experiments or without the aerial part of the plant exposed to naphthalene; NP: soil from planted pots exposed to naphthalene

16S rRNA amplicon sequencing showed that the most abundant phyla were Proteobacteria and Actinobacteriota in all samples, however, Proteobacteria were more abundant in clover experiments than in *Aeonium* while Actinobacteriota were more abundant in *Aeonium* than in clover. A slight increase in Proteobacteria (mainly in Gammaproteobacteria) was observed in samples exposed to naphthalene both in *Aeonium* and *Trifolium* experiments (Fig. 5, Suppl. Fig. 2).

The top genera identified in *Aeonium* experiments represented around 40% of the total sequences identified in soils and 45% of all the genera identified in soil with plants (Suppl. Table 3). Among them *Streptomyces* (belonging to the Actinobacteriota phylum) was the most abundant genus in all samples representing around 19–22% of the bacteria (Suppl. Table 3). Other abundant genera in all samples were members of the Actinomycetota phylum, *Kribbella* (2–3%) and *Actinoallomurus* (1.4–2.7%), of the Alphaproteobacteria order, *Pseudolabrys* (1.7–2%), and of the Chloroflexota phylum, *Ktedonobacter* (2.7–3.6%) and of the Gammaproteobacteria order, *Pseudomonas* (1–10%). *Pseudomonas* was the only group which increased its percentage in the presence of naphthalene regardless of the presence/absence of plants (Table 1). In soil samples (without *Aeonium*), *Massillia* increased its abundance in the presence of naphthalene while the *Burkholderia* group also increased but in the *Aeonium* planted-systems (Table 1). The presence of bacteria of the group *Escherichia-Shigella* declined in response to naphthalene, drastically in planted soils and slightly in bulk soils (Table 1), indicating that this group of bacteria was sensitive to this contaminant. *Flavobacterium* also decreased its proportions in the presence of naphthalene in systems with *Aeonium* plants while *Rhodococcus* decreased in soil samples with naphthalene (Table 1). *Novosphingobium,* although more abundant in planted soils than in bulk soils, also decreased in the presence of naphthalene in the soil (Table 1).

**Table 1:** Variations in the percentages of sequences assigned to the top genera in *Aeonium* experiments

	log_2_(SC/SN)	log_2_(PC/PN)	log_2_(SC/PC)	log_2_(SN/PN)
*Burkholderia-Caballeronia-Paraburkholderia*	0.82	− **1.44**	1.45	-0.81
*Pseudomonas*	− **2.06**	− **1.47**	− **1.83**	− 1.23
*Streptomyces*	0.14	− 0.18	0.20	− 0.12
*Rhodococcus*	3.08	0.90	3.55	1.37
*Escherichia-Shigella*	0.83	5.86	− **5.27**	-0.24
*Rhodanobacter*	− 0.04	0.82	− 0.11	0.75
*Dyella*	− 0.59	0.75	− 0.35	0.99
*Asticcacaulis*	0.40	0.52	0.17	0.28
*Massilia*	− **1.78**	0.68	0.15	2.62
*Chitinophaga*	-0.06	0.54	− 0.03	0.57
*Ktedonobacter*	0.14	0.40	− 0.16	0.11
*Actinoallomurus*	0.47	0.87	− 0.48	− 0.07
*Luteibacter*	− 0.46	0.23	0.27	0.96
*Staphylococcus*				
*Gemmatimonas*	0.35	− 0.01	0.35	− 0.01
*Flavobacterium*	0.49	2.69	− 1.32	0.88
*Novosphingobium*	1.49	− 0.17	− 0.27	− **1.93**
*Castellaniella*				
*Thermomonas*				
*Edaphobaculum*	− 0.62	− 0.09	0.20	0.73
*Kribbella*	0.03	− 0.49	0.57	0.06
*Chryseobacterium*			1.82	
*Nevskia*		− 0.27		
*Acidothermus*	0.28	0.24	0.36	0.33
*Chujaibacter*	− 0.03	− 0.30	0.57	0.30
*Arachidicoccus*	0.13	− 0.90	1.78	0.76
*Occallatibacter*	− 0.07	− 0.20	0.59	0.46
*Pseudolabrys*	0.04	− 0.25	0.19	− 0.11
*Bryobacter*	0.21	0.10	0.71	0.60

Increases (log_2_(condition1/condition2) > 1.3) are shown in underlined numbers and, decreases (log_2_(condition1/condition2) < -1.3) are shown in bolded numbers. SC: soil from experiments without the aerial part of the plant; PC: soil from experiments with *A. decorum*; NS: soil from experiments without the aerial part of the plant exposed to naphthalene; NP: soil from planted pots exposed to naphthalene

In *Trifolium repens* experiments, the top genera identified represented around 30–40% of the total sequences (Suppl. Table 3). *Streptomyces* (1–2%) and *Rhodanobacter* (2–12%) were the most abundant genera in all samples. *Devosia* sequences were the most abundant in non-planted soils both with or without exposure to naphthalene (Suppl. Table 3). Only *Solimonas* (belonging to Gammaproteobacteria) increased their abundance in response to naphthalene both in non-planted and planted soils (Table 2). There was an increase of Gamma-proteobacteria related to *Pantoea*, *Dyella* and of Bacteroidota-related *Arachidicoccus* in planted systems exposed to naphthalene, and a decrease in the Bacteroidetes genus, *Fluviicola,* and Betaproteobacteria *Massilia*. *Methanosarcina* increased their population in response to the contaminant in non-planted soil (Table 2).

**Table 2:** Variations in the percentages of sequences assigned to the top genera in clover experiments

	log_2_(SC/SN)	log_2_(PC/SN)	log_2_(SC/PC)	log_2_(SN/PN)
*Rhodanobacter*	− 0.04	− 1.07	− **1.48**	− **2.52**
*Devosia*	0.13	0.40	3.09	3.36
*Acidothermus*	0.73	0.10	− 1.41	− **2.04**
*Burkholderia-Caballeronia-Paraburkholderia*	− 0.46	− 0.57	− **3.81**	− **3.92**
*Solimonas*	− **5.70**	− **8.09**	3.88	1.49
*Nocardioides*	0.73	0.17	1.52	0.96
*Opitutus*	− 0.73			
*Lacunisphaera*	− 0.45	0.07	5.75	6.27
*Phenylobacterium*	− 0.20	0.92	3.17	4.29
*Castellaniella*	0.49	0.46	− **3.23**	− **3.26**
*Streptomyces*	0.38	− 0.82	0.95	− 0.25
*Unidentified_Candidatus_Jorgensenbacteria*		0.33		
*Dongia*	0.13	0.41	3.68	3.96
*Occallatibacter*	0.74	0.16	− **7.12**	− **7.70**
*Pantoea*		− **2.97**	− **7.15**	
*Pseudolabrys*	0.01	0.32	0.41	0.72
*Mucilaginibacter*	0.16	0.32	4.30	4.46
*Asticcacaulis*	− 0.39	0.19	0.16	0.75
*unidentified_Pedosphaeraceae*	− 0.29	0.06	− 0.94	− 0.59
*Dyella*	0.19	− **2.16**	0.85	− **1.51**
*Phaselicystis*	0.01			
*Methanosarcina*	− **2.50**			6.48
*Bryobacter*	− 0.22	0.48	− **2.49**	− **1.78**
*Massilia*	0.88	2.37	− **3.53**	− **2.04**
*Luteimonas*	− 0.11	0.75	4.29	5.15
*Arachidicoccus*	− 0.17	− **2.52**	− 1.22	− **3.57**
*Fluviicola*	0.37	4.20	− **4.17**	− 0.33
*Sphingomonas*	− 0.08	0.21	1.53	1.82

Increases (log_2_(condition1/condition2) > 1.3) are shown in underline numbers and, decreases (log_2_(condition1/condition2) < -1.3) are shown in bolded numbers. SC: bulk soils; PC: soils with clover; SN: bulk soils exposed to naphthalene; PN: soil from planted pots exposed to naphthalene

The changes at the genus level between planted and non-planted clover systems were more pronounced than with *Aeonium*, as already revealed in the analysis of diversity. *Rhodanobacter*, *Burkholderia* group, *Castellaniella*, *Occallatibacter*, *Bryobacter*, and *Massilia* were more abundant in planted soil regardless of exposure to the contaminant, while *Devosia*, Solimonas, *Lacunispheaera*, *Phenylobacterium*, *Dongia*, *Mucilaginibacter, Luteimonas* and *Sphingomonas* were less present than in planted soils (Table 2).

Therefore, *Pseudomonas* in *Aeonium* systems and *Solimonas* when using *Trifolium*, were the only genera that incremented their numbers in the presence of naphthalene in vegetated or non-vegetated systems.

**Fig. 6:**
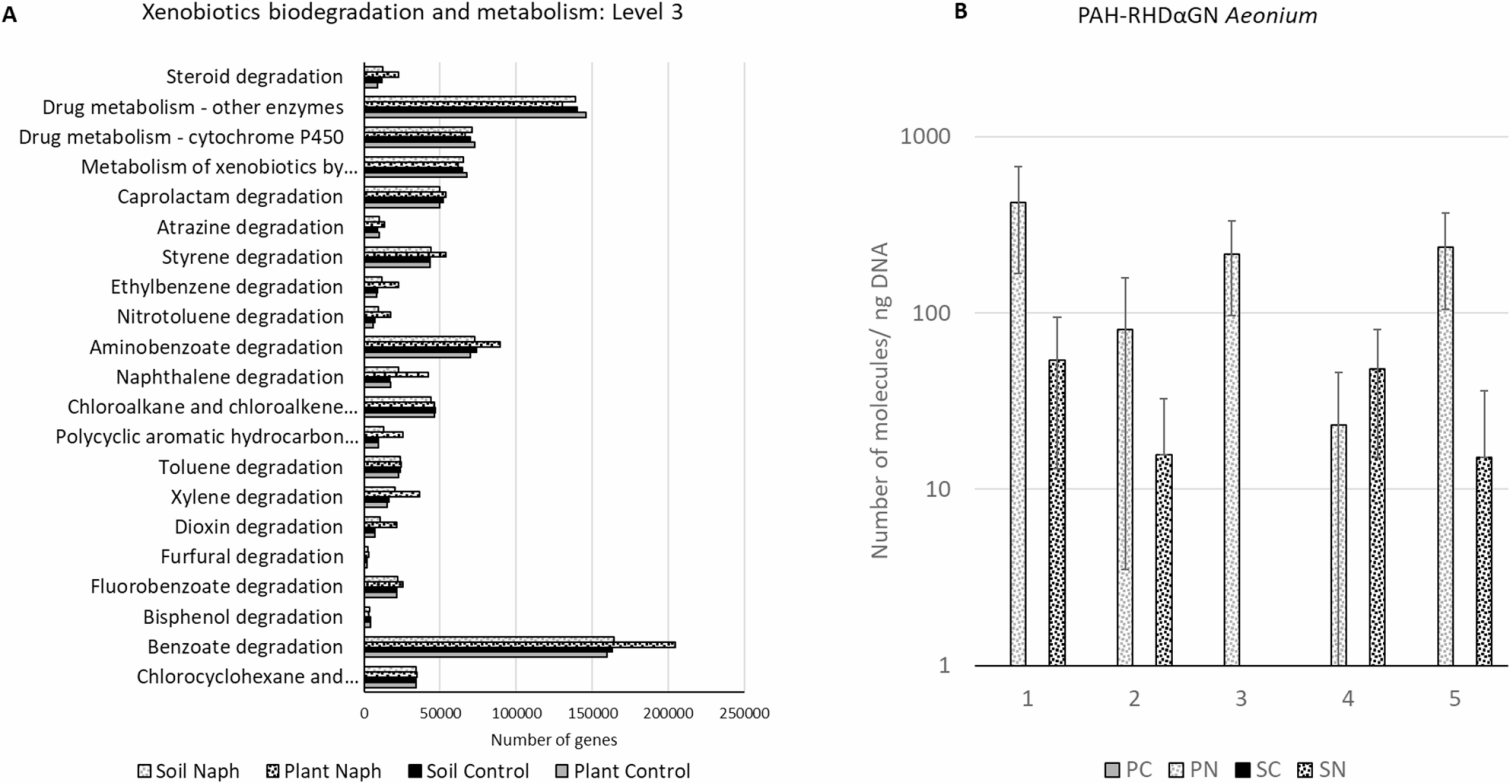
**A** KEGG functions related with xenobiotic degradation. **B** Detection of the subunit α of PAH dioxygenases in samples from *Aeonium*. No detection was observed in soil samples (SC) or soil with *Aeonium* (PC). PN: soil samples from experiments with plants and naphthalene; SN: soil samples with naphthalene. On the horizontal axis the results from 5 independent experiments are displayed. Bars represent the mean values of three qPCR replicates with error bars indicating the standard deviation

Given the positive results obtained with *A. decorum* systems, we carried out metagenomic analysis from soil samples of the *Aeonium* systems to identify key genes involved in response to naphthalene. We did not detect significant differences regarding the main biological processes (Suppl. Fig. 3A). However, there was an increase in the group of genes involved in functions related with xenobiotic biodegradation and metabolism in samples from plants with naphthalene (Suppl. Fig. 3B). Detailed analysis of the Unigenes related with the xenobiotics biodegradation revealed significant increases in categories such as steroid atrazine, styrene, ethylbenzene, nitrotoluene, aminobenzoate, naphthalene, xylene, dioxin, fluorobenzote and benzoate degradation genes in samples with plants and naphthalene (Fig. 6A). To further analyze the capacity of the different samples to degrade naphthalene, we estimated by qPCR, the number of PAH-ring hydroxylating dioxygenase alpha subunit of Gram negative bacteria (PAH-RHDα-GN) [26] as a proxy variable for the number of bacteria able to degrade naphthalene. In the *Aeonium* experiment, PAH-RHDα-GN genes were detected in systems containing naphthalene, with higher quantities observed in vegetated samples. In contrast, in the *Trifolium* experiments, the gene copy numbers were below the limit of detection (Fig. 6B).

### *Aeonium* gardens under environmental conditions did not show effects of naphthalene presence

**Fig. 7:**
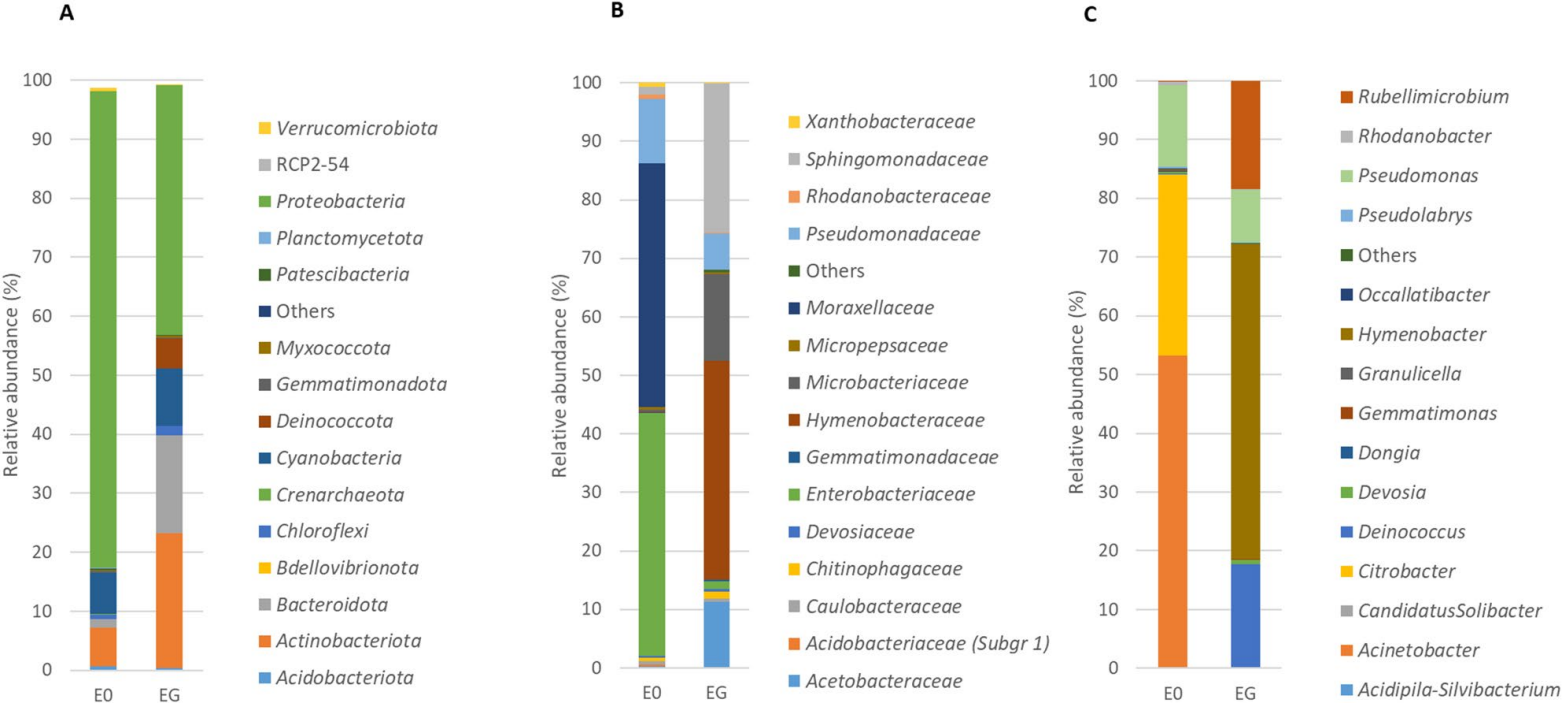
Bacterial diversity of epiphytic bacteria in *Aeonium decorum*. E0: samples at the initial time of the experiments. EG: samples from gardens after 6 months under atmospheric conditions. **A** Top 16 bacterial phyla of leaf samples from gardens. **B** Top 16 bacteria families of leaf samples (representing 76% of the E0 and 40% of the EG sequences). **C** Top 16 bacteria genera of leaf samples (representing 60% of E0 sequences and 27% of EG sequences)

Overall, our results suggested that the closed systems with *Aeonium* plants were able to dissipate higher quantities of gaseous naphthalene than similar systems with *Trifolium* and, although *Aeonium* was slightly more sensitive to the presence of the contaminant than *Trifolium*, the concentrations tested did not significantly alter the physiology of the plant. Furthermore, *Aeonium* plants recruited genes related with PAH degradation. Therefore, we decided to analyze artificial gardens with *Aeonium* plants. HPLC analysis of dust deposited on the surface of leaves did not reveal detectable concentrations of PAHs (naphthalene, pyrene, or phenanthrene). When we analyzed the diversity of epiphytic bacteria we observed a dramatic change in bacterial populations from the initial to the end point of the experiment. At the initial time point of the experiment there was a dominance of Gammaproteobacteria (mainly *Acinetobacter*) (Fig. 7A, C) while at the end of experiment the most abundant groups in gardens included Proteobacteria and Actinobacteriota. At the genus level, the most representative 15 genera at the starting point of the experiment represented 59% of the sequences, while in the gardens exposed for 6 months to environmental conditions, these genera represented only 27% of the variability (Suppl. Table 4). *Hymenobacter*, *Acidipila-Silvibacterium*, *Rubellimicrobium* and *Pseudomonas* were the most abundant genera in leaf samples after 6 months of exposure to environmental conditions (Fig. 7B).

In order to determine the capacity of the *Aeonium* leaves to support epiphytic bacteria capable of degrading contaminants, strains were isolated from enrichment cultures initiated from *Aeonium* leaves which are able to grow with phenanthrene as the sole carbon source. A number of colonies appeared (approx. 25 colonies) and two of these colonies have been further characterized and identified as members of the *Novosphingobium* genus (Segura et al. submitted for publication in *International Journal of Systematic and Evolutionary Microbiology*).

**Fig. 8:**
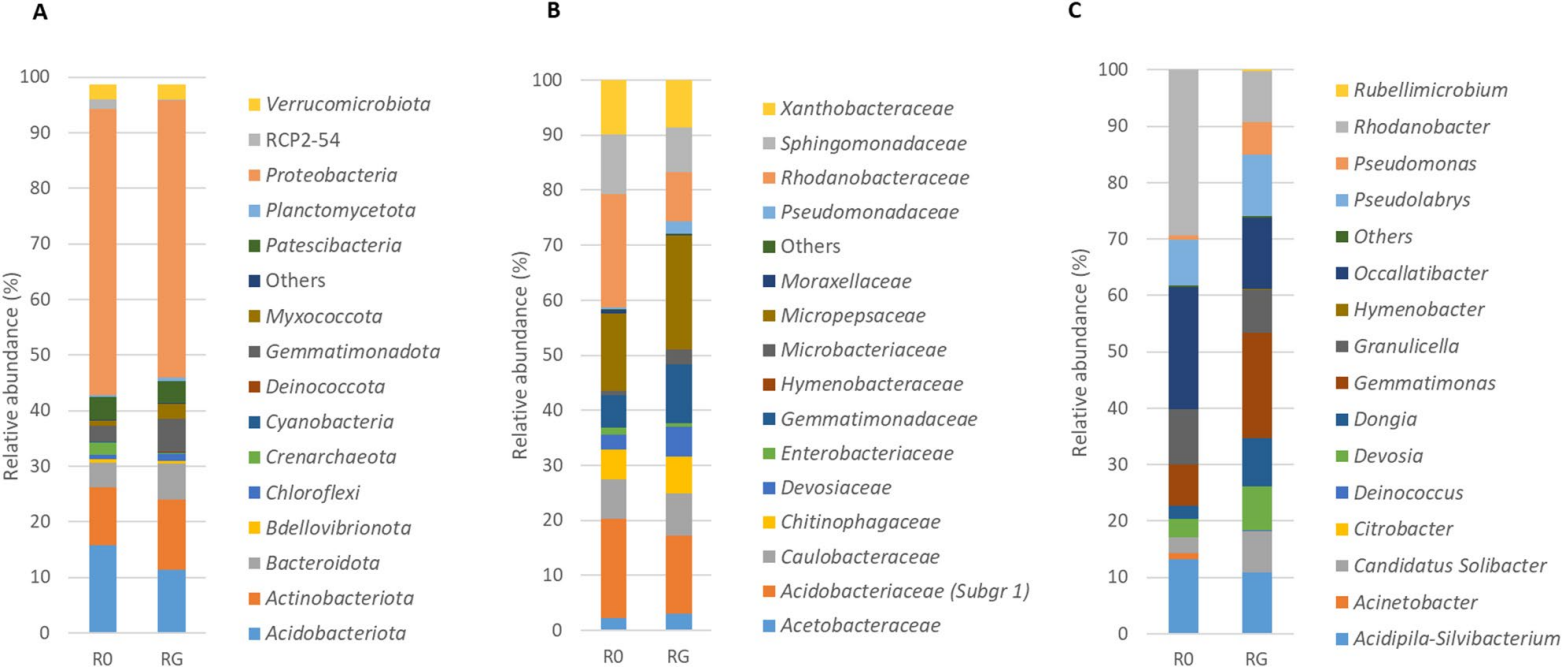
Bacterial diversity of rhizospheric bacteria in *A. decorum* gardens. R0: samples at the initial time of the experiment; RG: samples from gardens after 6 months under atmospheric conditions. **A** Top 15 bacterial phyla of soil samples. **B** Top 15 bacterial families of soil samples (that represented 43% of the sequences in R0 and 47% in RG. **C** Top 15 bacterial genera of soil samples (that constituted 14% of the sequences in R0 and 18% in RG)

Analysis of the diversity of rhizospheric bacteria within these gardens showed that they underwent minimal changes, as phylum- or family-level abundances remained largely stable between samples taken at the initial and final times of the experiment (Fig. 8A, B).

The main 15 main genera in samples (Fig. 8C) represented around 14% of the total bacterial diversity in samples taken at the initial time of the experiment and 18% in samples after 6 months under environmental conditions (Suppl. Table 5). The genus *Gemmatimonas* increased in the rhizospheric soil when compared with soils at the initial time in the artificial gardens with decreases in *Rhodanobacter* and *Occallatibacter.* The genus *Pseudomonas* experienced a slight increase in numbers in the gardens after being outside for 6 months (Fig. 8C), however, although the number of 16S rRNA genes assessed by qPCR was around 1–5 × 10^4^ molecules per ng of soil DNA in all samples, we did not detect genes related with PAH dioxygenases using the PAH-RHDα-GN oligonucleotides.

## Discussion

The utilization of green infrastructures for the elimination of atmospheric contaminants has been studied mainly as green filters for air-borne particles [38, 39]. However, it is long-known that plants, in addition to their capacity to adsorb, absorb and metabolize contaminants, can attract contaminant-degrading genes towards their rhizosphere, increasing their number near their roots [40]. Naphthalene is one of the toxic compounds among the volatile organic compounds (VOCs) in the atmosphere [41]. To evaluate the effect of this compound on urban green infrastructures, we set up a system in which naphthalene was added in solid phase without any contact with soil or plants.

The presence of gaseous naphthalene in our experimental systems was perceived by *A. decorum* plants, slightly decreasing the photosynthetic efficiency of the PSII. *A. decorum*, classified as a shrub species of *Aeonium* [42], is able to resist high temperatures and low humidity, characteristics that make these plants adequate for being used in roof gardens in the Mediterranean climate [43]. Our results showed that *A. decorum* was able to dissipate higher amounts of naphthalene than clover and other Mediterranean plants under similar conditions. However, there could be many different reasons behind these results. Biomass, plant physiology, and microbial metabolism could influence naphthalene dissipation through absorption, adsorption and transformation. As shown in Fig. 1, plant biomass was higher in the *A. decorum* experiments than in other systems (e. g., those with clover), potentially influencing adsorption and absorption processes. Plant transpiration may also actively mobilize naphthalene to the soil, increasing its availability for microbes [44]. However, through metagenomic analysis and qPCR, we have demonstrated that this plant was able to recruit microorganisms carrying genes related to naphthalene biodegradation in its rhizosphere. Several processes may contribute to this recruitment, but our qPCR results (Fig. 6B), clearly indicated that naphthalene is one of the drivers for this enrichment in *Aeonium* experiments as PAH-related dioxygenases were only amplified in samples exposed to naphthalene. However, this was not sufficient when the systems were planted with clover, in which these genes could not be amplified. Roots, decomposing biomass, microorganisms and plant canopies emit VOCs to the atmosphere, which are crucial in plant–microbe interactions [45, 46]. Soil microbes can utilize VOCs as carbon sources and VOCs could induce certain microbial contaminant-degrading pathways.

Soil composition differed between the experiments, which may have influenced the enrichment of degrading strains. Nevertheless, both experiments identified abundant genera known for their PAH-degrading capabilities. Species belonging to the genera *Burkholderia* and *Massilia*, which were among the most abundant genera identified in both *A. decorum* and clover soils, are recognized as good PAH degraders [47–50]. *Massilia* was enriched in bulk soil with naphthalene in *Aeonium* but not in clover (Table 1). *Burkholderia* were more abundant in bulk soils and slightly increased by the presence of naphthalene in both the *A. decorum* and clover experiments. *Pseudomonas*, a well-known naphthalene degrader [51–54], increased in abundance in response to naphthalene, both in the presence and absence of *A. decorum* plants. However, in clover systems, the most significant change in response to naphthalene was the increase in *Solimonas.* Members of the *Solimonas* genus have been isolated from different environments such freshwater springs or soil [55, 56]. Although limited information is available about the contaminant biodegradative capacities of this bacterial genus, recently, a soluble di-iron monooxygenase that could play an important role in bioremediation has been characterized in *Solimonas soli* [57]. Our results suggest that members of this genus could be relevant for bioremediation of naphthalene in soils and as marker for the presence of naphthalene, however more experiments would be needed to assess the role of these bacteria in mitigating atmospheric contaminants.

Root exudate composition varies depending on many different factors, such as plant type, age, and physiological status. Root exudates influence PAHs bioavailability, microbial proliferation, and the expression of biodegradative genes [58]. While some studies correlate the presence of degradative genes with higher degradation rates, others have shown that ryegrass root exudates stimulate bacteria harboring PAH ring-hydroxylating-dioxygenase alpha subunit (PAH_RHDα) genes, but this enhancement did not show a correlation with phenanthrene elimination [59]. Thus, the relationship between the recruitment of degradative genes in the rhizosphere and biodegradation abilities remains controversial.

Despite extensive phytoremediation research over the past two decades, the specific mechanisms that drive the complex role of plant-microbes during the biodegradation of organic contaminants, are still not fully understood [60]. Our study is among the few that demonstrate the recruitment of potential degradative microbes and genes in the rhizosphere of plants exposed to atmospheric contaminants. The observed increase in naphthalene dissipation is likely due to the many different effects discussed above, and further analysis will be needed to determine the contribution of microbial species and degradative genes to mitigate this atmospheric contaminant.

By establishing gardens with *A. decorum* under environmental conditions, we found that urban naphthalene levels at the time and location of our experiments were not sufficient to be detected on leaves by chemical methods, nor to induce the biological indicators observed in our controlled experiments (e.g. increased in *Pseudomonas* abundance and detection of PAHs degradation genes). However, we demonstrated that *Aeonium* leaf surfaces harbored bacteria capable of degrading PAHs, confirming that *A. decorum* is equipped with microbes that can participate in bioremediation.

Interestingly, a high proportion of *Hymenobacter* sequences were found among the epiphytic bacteria under environmental conditions. *Hymenobacter* species have been isolated from diverse environments, including soil and plant leaves, and some species are highly resistant to UV-radiation or and heavy metals [61, 62]. Their abundance in *A. decorum* plants highlights the potential for identifying new epiphytic bacteria with tolerance to radiation and with the capacity to tolerate/degrade contaminants which could be used to create microbial of consortia for atmospheric contaminant mitigation.

## Conclusions

Overall, our results demonstrate that *Aeonium decorum* plants possess key traits that make it a promising candidate for use as a bioremediation agent in green infrastructures. *A. decorum* plants not only enhance the biofilter capacity of soils and plants, but also promote the accumulation of potential naphthalene degrading microbiota in their rhizosphere, and also harbor natural populations on its leaves able to degrade PAHs.

## Supplementary Information

Below is the link to the electronic supplementary material.

Supplementary Material 1

## Data Availability

The datasets supporting the conclusions of this article are included within the article and its supplementary files. Raw sequencing data are available in the SRA repository, and will be made public if the manuscript is accepted. Access for reviewers have been granted and the corresponding links are included in the cover letter to editor.
