# Supplementary material for: Aeonium decorum as a microbial recruitment platform for atmospheric polycyclic aromatic hydrocarbons mitigation in urban gardens
Source: Environ Microbiome. 2026 May 31;21:95. doi: 10.1186/s40793-026-00914-7 (PMC13440189; doi:10.1186/s40793-026-00914-7)
Supplement: Supplementary file 1 [file 40793_2026_914_MOESM1_ESM.docx]

**Supplementary material**

***Aeonium decorum* as a microbial recruitment platform for atmospheric polycyclic aromatic hydrocarbons mitigation in urban gardens**

**Felix Velando, Lázaro Molina, Irene Hurtado, Pieter van Dillewijn and Ana Segura***

Environmental Protection Department, Estación Experimental del Zaidín, Consejo Superior de Investigaciones Científicas, C/ Profesor Albareda 1, 18008-Granada, Spain.

**Author for corresponding:** Ana Segura

[ana.segura@eez.csic.es](mailto:ana.segura@eez.csic.es)

**Supplementary Figure 1:** PCoA analysis of the microbial diversity of soil samples from S: soil without plants, P: soil with plants; SN: soil without plants and with naphthalene; PN: soil with plants and naphthalene, in experiments with *Aeonium decorum* (A) and *Trifolium repens* (B).

**Supplementary Figure 2** Top-ten bacterial Class components in the two experimental set-up, systems with *A. decorum* (left) and systems with clover (right). SC: soil from experiments non-planted or without the aerial part of the plant; PC: soil from experiments with plants; NS: soil from non-planted or without the aerial part of the plant exposed to naphthalene; NP: soil from planted pots exposed to naphthalene.

**Supplementary Figure 3**: Metagenome analysis of functions in the different experimental setups. A) General functions, B) Metabolism.

**Supplementary Table 1**: Alpha Diversity *p*-Values Summary

# ***Aeonium***

| Metric | Soil *vs* plant | Soil *vs* Soil plus naphthalene | Plant *vs* plant with natphthalene |
| --- | --- | --- | --- |
| Observed | 0.4418 | 0.6857 | 0.8857 |
| Shannon | 0.3282 | 0.8857 | 1 |
| Simpson | 0.1605 | 1 | 0.6857 |
| InvSimpson | 0.1605 | 1 | 0.6857 |
| Fisher | 0.6454 | 0.8857 | 0.4857 |

# ***Trifolium***

| Metric | Soil *vs* plant | Soil *vs* Soil plus naphthalene | Plant *vs* plant with naphthalene |
| --- | --- | --- | --- |
| Observed | 0.0001 | 0.6857 | 0.0286 |
| Shannon | 0.0001 | 1 | 0.1143 |
| Simpson | 0.0830 | 0.8857 | 0.1143 |
| InvSimpson | 0.0830 | 0.8857 | 0.1143 |
| Fisher | 0.0001 | 0.6857 | 0.0286 |

**Supplementary Table 2**: PERMANOVA Results Summary

# ***Aeonium***

| Comparison | Df | Sum of Squares | R² | F-value | p-value | Significance |
| --- | --- | --- | --- | --- | --- | --- |
| Soil vs Plant | 3 | 0.276 | 0.21533 | 1.0977 | 0.251 | ns |
| Residual | 12 | 1.0057 | 0.78467 |  |  |  |
| Total | 15 | 1.2817 | 1.0 |  |  |  |
| Soil vs Soil with naphthalene | 1 | 0.04443 | 0.10828 | 0.7285 | 0.865 | ns |
| Residual | 6 | 0.36592 | 0.89172 |  |  |  |
| Total | 7 | 0.41035 | 1.0 |  |  |  |
| Plant vs Plant with naphthalene | 1 | 0.1204 | 0.15837 | 1.1291 | 0.335 | ns |
| Residual | 6 | 0.63982 | 0.84163 |  |  |  |
| Total | 7 | 0.76021 | 1.0 |  |  |  |

# ***Trifolium***

| Comparison | Df | Sum of Squares | R² | F-value | p-value | Significance |
| --- | --- | --- | --- | --- | --- | --- |
| Soil vs Plant | 3 | 2.9779 | 0.79359 | 15.379 | 0.001 | *** |
| Residual | 12 | 0.7745 | 0.20641 |  |  |  |
| Total | 15 | 3.7524 | 1.0 |  |  |  |
| Soil vs Soil with naphthalene | 1 | 0.09287 | 0.18338 | 1.3473 | 0.035 | * |
| Residual | 6 | 0.41355 | 0.81662 |  |  |  |
| Total | 7 | 0.50642 | 1.0 |  |  |  |
| Plant vs Plant with naphthalene | 1 | 0.11203 | 0.23684 | 1.862 | 0.06 | . |
| Residual | 6 | 0.36099 | 0.76316 |  |  |  |
| Total | 7 | 0.47302 | 1.0 |  |  |  |

**Supplementary Table 3:** Percentage of the most abundant genera in soils from *Aeonium* and clover systems. The most abundant genera are in bold. S: bulk soil samples; P: planted-soil samples; C: control samples without naphthalene; N: samples exposed to naphthalene.

|  | *Aeonium* | | | |  |  | Clover | | | |
| --- | --- | --- | --- | --- | --- | --- | --- | --- | --- | --- |
|  | SC | SN | PC | PN |  |  | SC | SN | PC | PN |
| *Burkholderia-Caballeronia-Paraburkholderia* | 1.04 | 0.59 | 0.38 | 1.03 |  | ***Rhodanobacter*** | **2.18** | **2.12** | **12.44** | **5.92** |
| ***Pseudomonas*** | **1.04** | **4.35** | **3.69** | **10.22** |  | *Devosia* | 7.52 | 8.22 | 0.73 | 0.97 |
| ***Streptomyces*** | **21.87** | **19.84** | **19.02** | **21.52** |  | *Acidothermus* | 1.41 | 2.34 | 5.79 | 6.23 |
| *Rhodococcus* | 0.18 | 0.02 | 0.02 | 0.01 |  | *Burkholderia-Caballeronia-Paraburkholderia* | 0.22 | 0.16 | 3.37 | 2.26 |
| *Escherichia-Shigella* | 0.13 | 0.07 | 4.91 | 0.08 |  | *Solimonas* | 1.92 | 0.04 | 0.68 | 0.00 |
| *Rhodanobacter* | 1.17 | 1.20 | 1.26 | 0.71 |  | *Nocardioides* | 1.33 | 2.20 | 0.68 | 0.77 |
| *Dyella* | 0.18 | 0.28 | 0.23 | 0.14 |  | *Opitutus* | 2.49 | 1.50 | 0.00 | 0.00 |
| *Asticcacaulis* | 0.80 | 0.61 | 0.71 | 0.50 |  | *Lacunisphaera* | 2.01 | 1.47 | 0.03 | 0.03 |
| *Massilia* | 0.12 | 0.40 | 0.10 | 0.07 |  | *Phenylobacterium* | 2.50 | 2.18 | 0.13 | 0.24 |
| *Chitinophaga* | 0.06 | 0.06 | 0.06 | 0.04 |  | *Castellaniella* | 0.12 | 0.18 | 1.19 | 1.65 |
| ***Ktedonobacter*** | **3.19** | **2.91** | **3.56** | **2.70** |  | ***Streptomyces*** | **1.57** | **2.04** | **1.86** | **1.06** |
| ***Actinoallomurus*** | **1.96** | **1.42** | **2.73** | **1.49** |  | *unidentified_Candidatus_*  *Jorgensenbacteria* | 0.00 | 0.00 | 1.43 | 1.80 |
| *Luteibacter* | 0.27 | 0.37 | 0.22 | 0.19 |  | *Dongia* | 1.82 | 1.99 | 0.12 | 0.16 |
| *Staphylococcus* | 0.00 | 0.01 | 0.06 | 0.00 |  | *Occallatibacter* | 0.01 | 0.01 | 1.58 | 1.76 |
| *Gemmatimonas* | 0.29 | 0.23 | 0.23 | 0.23 |  | *Pantoea* | 0.00 | 0.00 | 1.45 | 0.18 |
| *Flavobacterium* | 0.15 | 0.11 | 0.38 | 0.06 |  | *Pseudolabrys* | 1.73 | 1.74 | 1.05 | 1.31 |
| *Novosphingobium* | 0.02 | 0.01 | 0.02 | 0.02 |  | *Mucilaginibacter* | 1.16 | 1.30 | 0.05 | 0.07 |
| *Castellaniella* | 0.00 | 0.00 | 0.00 | 0.00 |  | *Asticcacaulis* | 1.61 | 1.23 | 0.96 | 1.10 |
| *Thermomonas* | 0.00 | 0.00 | 0.00 | 0.00 |  | *unidentified_Pedosphaeraceae* | 0.62 | 0.50 | 0.93 | 0.97 |
| *Edaphobaculum* | 0.20 | 0.31 | 0.18 | 0.19 |  | *Dyella* | 0.52 | 0.60 | 1.48 | 0.33 |
| ***Kribbella*** | **3.05** | **2.99** | **2.05** | **2.88** |  | *Phaselicystis* | 1.10 | 1.11 | 0.00 | 0.00 |
| *Chryseobacterium* | 0.01 | 0.00 | 0.00 | 0.00 |  | *Methanosarcina* | 0.51 | 0.09 | 0.01 | 0.00 |
| *Nevskia* | 0.00 | 0.00 | 0.01 | 0.01 |  | *Bryobacter* | 0.26 | 0.23 | 0.91 | 1.27 |
| *Acidothermus* | 0.77 | 0.63 | 0.60 | 0.50 |  | *Massilia* | 0.04 | 0.07 | 0.15 | 0.77 |
| *Chujaibacter* | 0.30 | 0.31 | 0.20 | 0.25 |  | *Luteimonas* | 1.22 | 1.13 | 0.03 | 0.06 |
| *Arachidicoccus* | 0.03 | 0.03 | 0.01 | 0.02 |  | *Arachidicoccus* | 0.07 | 0.07 | 0.88 | 0.15 |
| *Occallatibacter* | 0.40 | 0.42 | 0.26 | 0.30 |  | *Fluviicola* | 0.02 | 0.03 | 0.02 | 0.46 |
| ***Pseudolabrys*** | **1.91** | **1.85** | **1.68** | **2.00** |  | *Sphingomonas* | 0.93 | 0.88 | 0.26 | 0.30 |
| *Bryobacter* | 1.15 | 1.00 | 0.66 | 0.70 |  |  |  |  |  |  |
| **Others** | **59.73** | **59.99** | **56.71** | **54.19** |  | **Others** | **66.59** | **65.10** | **70.19** | **61.78** |

**Supplementary Table 4**: Mean abundance of sequences representing the most abundant genera in leaf samples from gardens. E0: samples at the initial time of the experiments. EG: samples from gardens after 6 months under atmospheric conditions.

| **E0** | | **EG** | |
| --- | --- | --- | --- |
| **Taxon_Grouped** | **Mean_Abundance** | **Taxon_Grouped** | **Mean_Abundance** |
| *Acidipila-Silvibacterium* | 0.00059229 | *Acidipila-Silvibacterium* | 0 |
| *Acinetobacter* | 0.31677187 | *Acinetobacter* | 0.00035147 |
| *CandidatusSolibacter* | 0.00021999 | *Candidatus Solibacter* | 3.5353E-05 |
| *Citrobacter* | 0.18355812 | *Citrobacter* | 0.00012075 |
| *Deinococcus* | 0.00065998 | *Deinococcus* | 0.04804337 |
| *Devosia* | 0.00170917 | *Devosia* | 0.00190441 |
| *Dongia* | 0.00155687 | *Dongia* | 0.00033117 |
| *Gemmatimonas* | 0.00013538 | *Gemmatimonas* | 0.00010492 |
| *Granulicella* | 0.00057536 | *Granulicella* | 0 |
| *Hymenobacter* | 0.00084612 | *Hymenobacter* | 0.14691256 |
| *Occallatibacter* | 0.00037229 | *Occallatibacter* | 1.7852E-05 |
| Others | 0.0003224 | Others | 0.00067756 |
| *Pseudolabrys* | 0.00174302 | *Pseudolabrys* | 0.0003257 |
| *Pseudomonas* | 0.08268323 | *Pseudomonas* | 0.02445555 |
| *Rhodanobacter* | 0.00329988 | *Rhodanobacter* | 0.00037057 |
| *Rubellimicrobium* | 0.00137072 | *Rubellimicrobium* | 0.0504727 |
| **TOTAL** | **0.59641669** | **TOTAL** | **0.27412394** |

**Supplementary Table 5**: Mean abundance of sequences representing the most abundant genera in rhizospheric samples from gardens. E0: samples at the initial time of the experiments. EG: samples from gardens after 6 months under atmospheric conditions.

| **E0** | | **EG** | |
| --- | --- | --- | --- |
| **Taxon_Grouped** | **Mean_Abundance** | **Taxon_Grouped** | **Mean_Abundance** |
| *Acidipila-Silvibacterium* | 0.01789471 | *Acidipila-Silvibacterium* | 0.01924586 |
| *Acinetobacter* | 0.00154592 | *Acinetobacter* | 2.04E-05 |
| Candidatus *Solibacter* | 0.00371783 | Candidatus *Solibacter* | 0.01285403 |
| *Citrobacter* | 0 | *Citrobacter* | 0 |
| *Deinococcus* | 0 | *Deinococcus* | 0.00027146 |
| *Devosia* | 0.00456468 | *Devosia* | 0.01374144 |
| *Dongia* | 0.00325079 | *Dongia* | 0.01523852 |
| *Gemmatimonas* | 0.0099447 | *Gemmatimonas* | 0.03311083 |
| *Granulicella* | 0.01315285 | *Granulicella* | 0.01344654 |
| *Hymenobacter* | 0 | *Hymenobacter* | 0.00017904 |
| *Occallatibacter* | 0.02949551 | *Occallatibacter* | 0.02231311 |
| Others | 0.00056142 | Others | 0.00053705 |
| *Pseudolabrys* | 0.0109022 | *Pseudolabrys* | 0.01937197 |
| *Pseudomonas* | 0.00114918 | *Pseudomonas* | 0.01017527 |
| *Rhodanobacter* | 0.03987954 | *Rhodanobacter* | 0.01602376 |
| *Rubellimicrobium* | 0 | *Rubellimicrobium* | 0.00031146 |
| **TOTAL** | **0.13605934** | **TOTAL** | **0.17684072** |
